# Estrogen-Gut-Brain Axis: Examining the Role of Combined Oral Contraceptives on Mental Health Through Their Impact on the Gut Microbiome

**DOI:** 10.7759/cureus.81354

**Published:** 2025-03-28

**Authors:** Abigail Zim, Ajay Bommareddy

**Affiliations:** 1 Department of Pharmacy, Nesbitt School of Pharmacy, Wilkes-Barre, USA; 2 Department of Biomedical Science, Charles E. Schmidt College of Medicine, Florida Atlantic University, Boca Raton, USA

**Keywords:** combined oral contraceptives, estrogen-gut axis, gut-brain axis, gut flora, gut microbiome

## Abstract

Combined oral contraceptives (COCs) possess the ability to alter the normal composition of the gut microbiome and the permeability of the gastrointestinal (GI) tract, which may cause both gut-related and non-gut-related complications. The gut-estrogen axis examines the relationship between estrogens (particularly the active form, estradiol) and the gastrointestinal system and can be attributed to the maintenance of the estrobolome and circulating estradiol levels. The gut-brain axis involves the relationship between the brain and the gastrointestinal system and can be attributed to the gut microbiome in relation to the enteric nervous system (ENS) and serotonin levels. Overall, the introduction of exogenous hormones into an endogenous environment alters the normal balance of both hormones and bacteria. Currently, there is a gap in knowledge regarding the link between COCs and mental health complications such as anxiety and depression, and the diversity in these complications may be related to different types of COCs, their composition, and variations in study populations. This article reviews existing evidence from animal and human studies on the role of COCs in the development of mental health issues through their impact on the gut microbiome.

## Introduction and background

Oral hormonal contraception is the most commonly prescribed method of birth control [1]. An estimated 25% of women of reproductive age (15-44 years) report that oral hormonal contraception is their preferred method of birth control. Oral hormonal contraception comes in two forms: combined oral contraceptives (COCs) and progestin-only pills (POPs). Progesterone is a naturally occurring endogenous hormone that plays a major role in the female reproductive system, whereas progestin is a synthetic hormone used in contraceptive pills to mimic the function of progesterone. Of the two forms, COCs are prescribed frequently for the prevention of unwanted pregnancy. They are also used for the treatment of acne, fibroids, and pain associated with endometriosis and for the regulation of menstrual symptoms (i.e., bleeding, pain, and migraines) [1]. Along with mild side effects including nausea, headaches, abdominal cramping, and breakthrough bleeding, certain adverse effects associated with COCs include the risk of venous thromboembolism (VTE), particularly during the first year of initiation, and the risk increases with high doses of ethinyl estradiol and third- and fourth-generation progestin [1]. In addition, the risk of ischemic stroke or myocardial infarction increases with higher doses of estrogen.

Drospirenone, a POP, has anti-mineralocorticoid activity that might cause hyperkalemia in high-risk individuals, and caution should be exercised before initiating it. Studies have also suggested that the long-term use of COCs may be associated with gastrointestinal (GI) and central nervous system (CNS) complications, but the source of this association is not fully understood. The role of COCs on mood disorders may be attributed to the concentration and the type of progestin analog that is included in the product. Endogenous progesterone in the presence of estrogen is known to reduce the number of estradiol (E2) receptors and decrease the estradiol function that may be sufficient to produce a negative effect on mood in long-menopausal-duration women. However, the addition of progestin in short-menopausal-duration women who have higher circulating pretreatment estradiol and estradiol receptors may not be sufficient to adversely affect mood states. In addition, external progestins as a part of COCs increase the levels of monoaminoxidase that degrade serotonin concentrations and thus potentially precipitate irritability and mood disorders [2]. Another possible mechanism associated with COCs in mediating their effects on brain function may be the gut microbiome. Gut dysbiosis, referred to as a disruption of gut flora, has been linked to several metabolic, gastrointestinal, and inflammatory diseases [3]. Studies suggested that the use of COCs may be associated with the dysregulation of the gut microbiome, which over time has become a relatively well-established factor at play in the development of CNS complications. Most recently, COCs have been shown to reduce diversity and bacterial richness in the gut, and the users displayed reduced alpha bacteria diversity but not beta diversity when compared to non-users [4].

Several recent studies have shown that the likelihood of experiencing depressive symptoms or clinically relevant depression is higher in adolescent women using COCs compared to the same population group not using COCs. This article is aimed at understanding various factors that have a role in contributing to the complications and focuses on the existing literature on the emerging concept of the role COCs may play in CNS complications through their impact on the composition and diversity of the gut microbiome. In this review, we compiled information by performing a literature search using the keywords “Gut microbiome”, “COCs and depression”, “COCs and gut microbiome”, and “Estrogen gut brain axis” in PubMed and Google Scholar.

## Review

Gut microbiome

The gut microbiome is a diverse population of microorganisms living within the tract [5]. The development of the gut microbiome in a person begins from the moment of their birth, due to exposure to a variety of microorganisms during vaginal delivery. The gut microbiome subsequently grows and diversifies as life progresses and as an individual gets exposed to diverse microorganisms from consumed food and their surrounding environment. It is well-documented that the delivery method of newborns could influence microbiota’s differentiation and development, which ultimately contribute to differences in normal physiological processes and disease predisposition. While neonates are exposed to a variety of microbes provided by the mother during and after passage through the birth canal, babies delivered through cesarean section have limited exposure to the microbes that have been linked to the differences in their intestinal microbiota [6]. The collaboration relationships with intestinal bacteria are known to influence various functions in the body, including energy balance, resistance to pathogen colonization, the metabolism of xenobiotics, and the maturation of the intestine and immune system.

The most common bacterial phyla found within the gut include Firmicutes and Bacteroidetes, which together make up 90% of the gut microbiome composition. The microbiome extends itself all throughout the tract, with bacteria such as *Lactobacillus* and Enterobacteriaceae being found in the small intestine and bacteria such as Bacteriaceae, Prevotellaceae, Rikenellaceae, Lachnospiraceae, and Ruminococcaceae being found in the colon [7]. Most of the bacteria that make up the composition of the gut flora have a symbiotic relationship, wherein the bacteria benefit both themselves and the host in which they reside, in this case the human tract. However, certain bacterial strains within the gut are pathogenic and possess the potential to harm their host. In a healthy individual, the commensal bacteria have control over the microbiome and help to negate the effects of the pathogenic bacteria and promote normal function. While commensals mostly promote health, depending on the host body’s functioning and internal environment, they may also become harmful. Therefore, the introduction through probiotics and the sustenance of various types of commensal bacteria is important for developing and maintaining a diverse gut microbiome composition and, as a result, a healthy functioning gut [8,9].

Furthermore, a diverse gut microbiome extends beyond helping just gastrointestinal function and is described as “intimately connected to human physiology,” since it aids in all bodily systems and functions [10]. If the bacterial balance in our gut is disrupted, it leads to increased intestinal permeability, referred to as a “leaky gut.” The development of a leaky gut opens the door for pathogenic bacteria to gain access and take control of the microbiome. As a result, there is an increased risk of developing gut-related and non-gut-related complications. Gut-related complications that can occur include functional diseases such as irritable bowel syndrome and inflammatory bowel diseases (IBD), including Crohn’s disease (CD). Non-gut-related complications that can occur include depression, obesity, and type II diabetes mellitus.

Drug-induced disruption of the gut microbiome is well-understood in the case of antibiotics. Broad-spectrum antibiotics exhibit either bacteriostatic or bactericidal activity against a wide variety of bacteria. While this is a benefit in the case of treating an infection empirically, it serves as a detriment to the composition of the gut microbiome since their capability to distinguish between symbiotic and pathogenic bacteria is insufficient. As a result, the antibiotics may unintentionally fight off some of the important bacteria found within our gut and pave the way for opportunistic pathogens to take over. A prime example of this interaction is seen in the case of a *Clostridium difficile *infection, an infection of the colon that causes severe watery diarrhea. The majority of *C. difficile* infections occur during or after treatment with a broad-spectrum antibiotic, such as clindamycin, due to the antibiotic’s disruption of the normal gut flora [11].

Aside from antibiotics, little focus is put into the consideration of other potential medications that may pose the same threat of disrupting the gut microbiome. In the current review, we focus on one such medication class, COCs, which appear to have the ability to disrupt the bacterial composition of the gut in a manner similar to that understood with antibiotics, albeit by a different mechanism. As evidence continues to support the impairment of gut microbiota composition and the implication of the gut-brain axis in depression, anxiety, and other psychiatric disorders, interventional approaches to improve gut microbiota balance, including probiotics, have been extensively researched to establish their usefulness. For example, the gut microbiota of patients with major depressive disorder (MDD) showed a decrease in the diversity of strains with an increase in proinflammatory mediators, cortisol levels, and also alterations in the metabolism of tryptophan. As reviewed by Alli et al., several studies suggest that the usage of probiotics that often consist of *Lactobacillus* and *Bifidobacterium* genera has been shown to suppress inflammation and confer physical and mental health benefits to the host [12]. In a rat maternal separation model (stress-related GI and mood disorder model), the chronic administration of the probiotic *Bifidobacterium infantis* resulted in the reversal of behavioral deficits, restoration of basal noradrenaline concentrations in the brain stem, and normalization of immune response [13]. Similarly, a different study employing a chronic mild stress mouse model of depression showed that a mixture of three probiotic strains (*Lactobacillus plantarum*, *Lactobacillus helveticus*, and *Bifidobacterium longum*) could alleviate chronic mild stress-induced anxiety and depressive-like behaviors in mice [14]. Most recently, a comprehensive review conducted by Merkouris et al., on the clinical trials from 2014 to 2023, indicated that the majority of the studies support the use of probiotics in alleviating depressive and anxiety symptoms, albeit the benefit appears to be more prominent in people with milder symptoms [15]. Despite these advances in their effectiveness and mechanism of action, further research is warranted to clearly establish the usefulness of probiotics as a stand-alone option or a complementary treatment combined with other therapeutic agents aimed at improving mental health and other psychiatric conditions.

Combined oral contraceptives (COCs)

**Figure 1:**
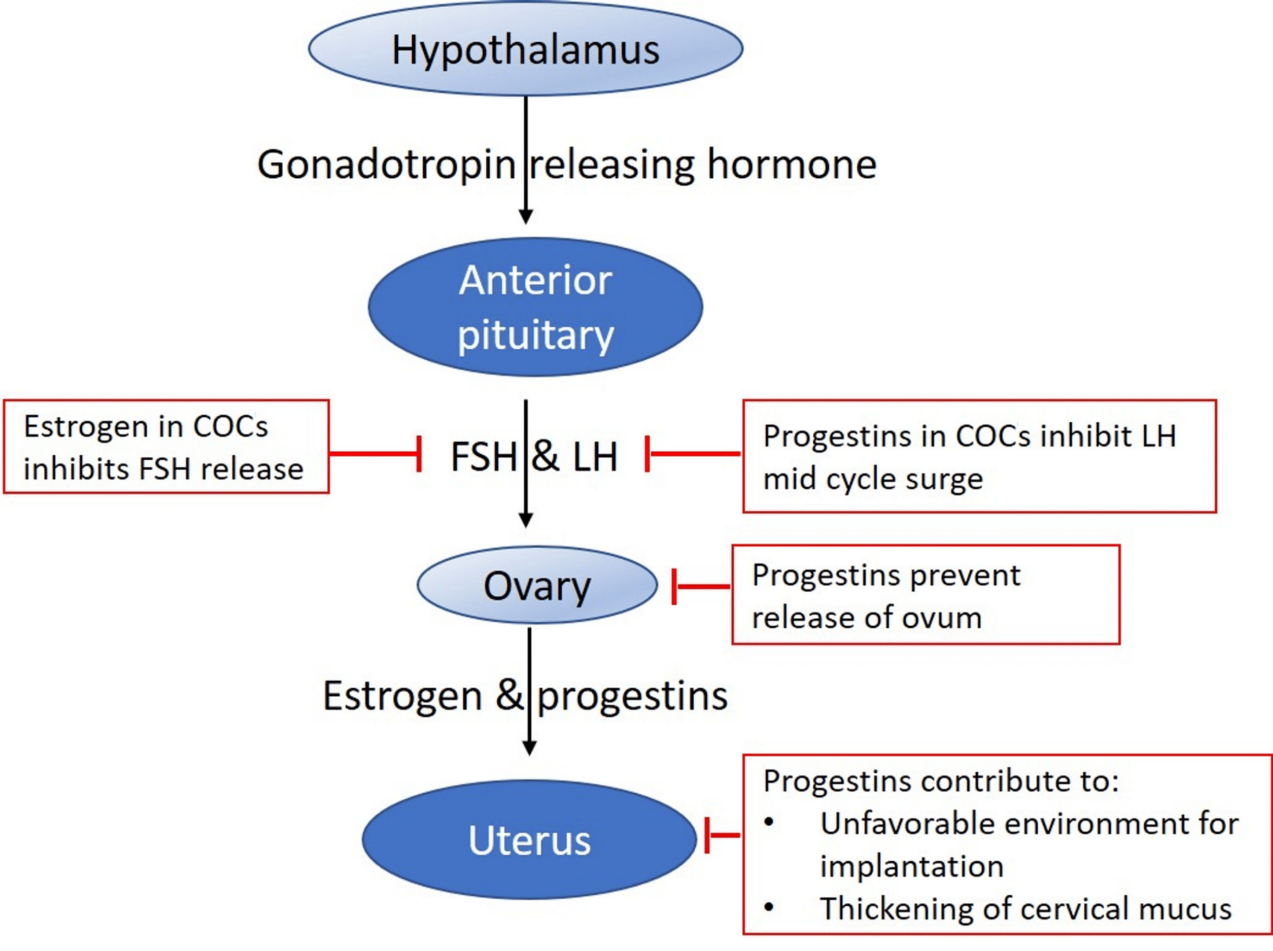
Pharmacology of combined oral contraceptives (COCs) Image conceived based on the information from Cooper et al. under the terms of the Creative Commons Attribution [1]. Not required to obtain permission FSH, follicle-stimulating hormone; LH, luteinizing hormone

To further understand the impact that combined oral contraceptives have on the gut microbiome, the mechanism by which these agents work on the human body must be discussed. COCs utilize the combined pharmacological action of two hormones, progestins and estrogen, in order to prevent pregnancy. Estrogen is primarily produced in the female gonads, ovaries, and also adrenal glands and adipose tissue and mainly consists of estrone (E1), estradiol (E2), and estriol (E3). E3 is the main metabolite of E1 and E2 synthesized by the ovaries, and E2 is the most metabolically active form, while E1 and E2 can be converted into each other [16,17]. The physiological effects of E2 are mediated through its interactions with two subtypes of intracellular receptors: estrogen receptor (ER) alpha and beta and the novel membrane-bound/nongenomic receptor G protein-coupled ER1. In addition to being expressed in the reproductive organs and other parts of the body, ERs are also expressed throughout the brain, including the hypothalamus, midbrain, and amygdala, all of which have been shown to modulate functions associated with the gastrointestinal system [16]. Progestins function through two mechanisms to inhibit ovulation and subsequently prevent pregnancy. Primarily, the introduction of exogenous progestins into the body induces a decrease in the release of gonadotropin-releasing hormone (GnRH) from the hypothalamus, which subsequently decreases the release of follicle-stimulating hormone (FSH) and luteinizing hormone (LH) from the anterior pituitary gland. The overall result is the inhibition of follicular development and a decrease in the amount of circulating estradiol. Secondarily, progestins prevent pregnancy by thickening the composition of the cervical mucus, making it too difficult for sperm cells to penetrate beyond the cervical and genital tracts. Additionally, without a sufficient level of circulating estradiol, the LH surge that is necessary for the follicle to be released cannot occur. As a result, pregnancy is prevented since ovulation cannot occur without a properly developed or released follicle. A summary of the pharmacology of combined oral contraceptives is illustrated in Figure 1.

Estrogen-gut axis

Now that the mechanism of action of COCs has been established, an important consideration when assessing their impact on the composition of the gut microbiome is the estrogen-gut axis. This axis refers to the interaction between the gut microbiome and estrogens. In a normal endogenous environment, the gut microbiome plays an important role in the regulation of circulating estradiol levels, as highlighted by the findings of a cross-sectional study including 25 men, seven postmenopausal women, and 19 premenopausal women, which suggested a link between gut microbiome composition and systemic estrogen concentrations [18,19]. The specific population of microorganisms within the gastrointestinal tract that has the potential to regulate estrogen levels is referred to as the estrobolome [10,19]. During estrogen metabolism, estrogens are conjugated in the liver via the addition of a glucuronic acid component, which allows for their excretion through the urine and feces [19,20]. However, bacteria within the estrobolome possess the beta-glucuronidase (*GUS*) gene, which encodes for the gut microbial beta-glucuronidase (gmGUS) enzyme [19]. This enzyme is vital in estrogen metabolism as it deconjugates estrogen into its active forms [19,20]. The gene that encodes for this enzyme was initially found in *Escherichia coli* and Enterobacteriaceae, both of which are commonly found within the normal flora of the gut [19]. Since then, the Human Microbiome Project GI database has compiled a list of bacterial phyla within the gut that possess the *GUS* gene, including but not limited to Bacteroidetes, Firmicutes, Verrucomicrobia, and Proteobacteria [18].

Once exogenous hormones are introduced into the body through COCs, the normal composition of the estrobolome is disrupted, and the aforementioned regulation of estradiol levels is disrupted. The role of exogenous hormones in gut-related complications is well-established. The earliest case reports suggesting a link between combined oral contraceptive use and gastrointestinal inflammation and dysfunction were from the late 1960s and early 1970s [21]. There have been a handful of studies examining the same correlation, and studies suggest a potential increased risk for the development or relapse of Crohn’s disease in women taking combined oral contraceptives. A meta-analysis published in 2008 found that the risk of developing Crohn’s disease (CD) increased significantly when COCs were used [22]. Furthermore, the risk significantly increased with the length of the use of COCs and became insignificant upon the discontinuation of COCs [22]. As noted earlier, CD is a functional gastrointestinal disorder that can arise as a complication of the disruption of normal gut flora composition. Based on the meta-analysis, it may be concluded that the correlation between the use of COCs and the development of CD could be linked to the impact of COCs on the gut microbiome [22].

A randomized controlled trial showed a statistically significant increase in the risk for relapse of Crohn’s disease for both past and current users, as compared to non-users. Forty-three percent of women who experienced CD relapse were current users of COCs, compared to 70% of women who had used COCs in the past and 27% of women who had never used COCs. The results were found to be significant even when adjusted for confounding variables, such as smoking status, and the authors concluded that the use of COCs may independently increase the risk of CD relapse [23]. A most recent nested study that examined the association between the types of hormonal contraception and the development of inflammatory bowel disease (IBD) showed that the use of COCs was associated with CD and ulcerative colitis. The authors found that the risk of CD increased among current users for second-generation COCs than newer agents when compared to non-users. However, there was no difference in CD risk for those using low-strength estrogen pills compared to standard-strength estrogen pills. In addition, progestin-only pills and parenteral contraceptive methods were not associated with an increased risk of CD compared to non-users [24]. These findings are particularly useful to women with a strong family history of IBD seeking contraception.

Additionally, the disruption of normal intestinal permeability is a major factor in the development of IBD and CD. The exact mechanism by which COCs may alter the disease progression is not completely understood. However, a number of hypotheses are biologically plausible. Notably, the introduction of exogenous estrogen into an endogenous environment through the consumption of COCs may lead to changes in intestinal permeability, which, as established earlier, leads to the development of a “leaky gut,” a major biological pathway involved in the pathogenesis of CD and the disruption of the normal gut flora. COCs alter the endogenous levels of hormones, particularly estrogen, and enhance the cellular proliferation and humoral immune system, which may alter disease progression. This observation is limited to estrogen-containing oral contraceptives (OC) and was further confirmed by a prospective study conducted by Khalili et al., where it was shown that the consistent and long-term use of COCs is associated with an increased risk of surgery among women with established CD [25]. In addition to the potential effect of COCs on intestinal barrier function and immune function, recent animal data have suggested that gut commensal microbes may modulate levels of endogenous testosterone, leading to the development and progression of autoimmune diseases. Therefore, these findings suggest a complex interaction between endogenous levels of sex hormones, immune function, and gut microbiome in regulating the development and progression of immune-mediated diseases.

Gut-brain axis

**Figure 2:**
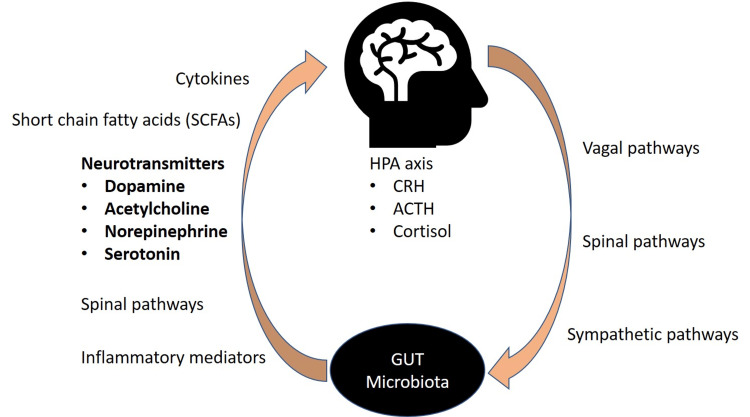
Gut-brain axis Communication between gut microbiota and the brain. Includes the vagus nerve, SCFAs, cytokines, inflammatory mediators (IL-6 and IL-1), and neurotransmitters. Image reproduced from Mudyanadzo et al., an open-access article under Creative Commons Attribution license [29] ACTH, adrenocorticotropic hormone; CRH, corticotropin-releasing hormone; HPA, hypothalamic-pituitary-adrenal; IL, interleukin

The gut-brain axis refers to the interaction between the gut microbiome and the brain that can occur via various mechanisms, as depicted in Figure 2. The bidirectional and collaborative interactions between the brain and the gut microbes are not fully understood but are based on the metabolic, neural, endocrine, and immunological pathways. The interactions include the hypothalamic-pituitary-adrenal (HPA) axis, immune mediators, endocrine signaling, and bacterial metabolites. Earlier studies have identified that neural communication between gut microbes and centrally mediated behavioral effects are carried out by the vagus nerve and that humans who underwent full truncal vagotomy are found to have a decreased risk of certain neurological disorders, such as Parkinson’s disease [26]. Similarly, a defective communication of the gut-brain axis along with other complications has been well-documented to be part of depression and various gastrointestinal pathologies, including IBS [27-29]. For example, inflammatory mediators, including interleukin-6 and interleukin-10, are at the forefront of intestinal inflammation, altering gut microflora and the permeability of gut mucosa.

The immune regulation of the HPA axis is mediated through the modification of cytokine secretion. Cortisol plays an important role in the endocrine pathway that regulates the gut-brain axis and modulates the immune system’s function by regulating the secretion of cytokines that not only act on the HPA axis but also alter the differentiation and physiological effects of the intestinal microbiota [30]. The altered HPA axis through inflammation and leaky gut induced by stress could play a role in neurotransmission and contribute to mood disorders, including depression. Microflora in the gut can produce a majority of the neurotransmitters found in the brain via the enteric nervous system (ENS), and hence, the ENS is often referred to as the “second brain.” There is evidence to suggest that the gut microbiota can alter the levels of neurotransmitter precursors, such as increased tryptophan levels by *Bifidobacterium infantis*, which ultimately influences central serotonin transmission [31]. The synthesis of other neurotransmitters, including dopamine, acetylcholine, and norepinephrine, is also produced by different bacterial species thriving in the gut. While the neurotransmitters synthesized in the gut may have a localized effect and limited influence on brain function, gut bacteria could impact the brain function indirectly through their metabolites including small-chain fatty acids (SFAs) such as butyrate, propionate, and acetate that could serve as epigenetic modulators in addition to inhibiting lipopolysaccharide (LPS)-induced inflammation and can suppress ongoing inflammation in the CNS [32,33].

Serotonin, in particular, has been a primary focus with regard to normal mental function. The serotonin hypothesis, which was coined in 1967 by a British psychiatrist named Alec Coppen, has suggested that deficiencies in serotonin levels and activity are observed in those suffering from major depressive disorder (MDD) [34]. This hypothesis has been and continues to be up for debate. A recent systematic review concluded that there simply has not been sufficient evidence over the years to support this hypothesis [35]. On the other hand, a different narrative review suggests that the available evidence offers a correlation between abnormalities in serotonin mechanisms and patients suffering from depression [34]. However, the authors do note that since correlation does not equal causation, it remains difficult to determine the true role of serotonin mechanisms in depression [34].

The gut microbiota also regulates the metabolism of glutamate, which in turn could influence the synthesis of gamma-aminobutyric acid (GABA). Studies have shown that gut microbiota produces GABA that reaches the CNS via GABA transporters expressed in the blood-brain barrier (BBB). In addition, metabolites such as acetate produced by microbiota are transferred across the BBB to the hypothalamus and enter the GABA neurological system. Dopamine is typically produced in the substantia nigra, ventral tegmental area, and hypothalamus and is released into the nucleus accumbens and the prefrontal cortex of the brain. Certain bacterial strains, including *Bacillus* and *Serratia* species, are shown to produce catecholamines (dopamine and norepinephrine) in the gut lumen of mice [36]. Similarly, histamine and glutamate synthesis are also achieved by certain gut bacteria species such as *Enterobacter* and *Lactobacillus* [37].

In a healthy individual, to maintain normal brain chemistry and mental function, the gut acts via neurotransmitters found within the ENS, such as serotonin. A diverse composition of bacteria within the gastrointestinal tract is essential for promoting the healthy function of the ENS and, subsequently, the brain. However, when the composition of the gut microbiome is disrupted, the balance of serotonin within the ENS is also disrupted, and as a result, mental health complications may arise. For example, in an animal study examining the association between the composition of the gut microbiome in postpartum female mice and depressive behaviors, the authors concluded that the disruption of the normal composition of the gut microbiome may manifest itself through mental health complications, as shown by the depressive-like changes in mood and behavior of the mice [38]. Similarly, another study revealed that dysbiosis mediated by antibiotics in mice contributed to an inflammatory state and depressive-type behavior that was reversed with the probiotic *Lactobacillus casei* [39]. A systematic review that compiled 19 years’ worth of evidence from 2000 to 2019 concluded that there is a correlation between having a decreased diversity in the bacterial makeup of the gut microbiome and being diagnosed with depression [40].

Estrogen-gut-brain axis

Although COCs are the most commonly prescribed method of birth control, many women discontinue taking them due to reported mood changes and feelings of depression. A prospective cohort study conducted in Denmark suggested that there is a “small but real” increase in the risk of developing depression while on any form of hormonal birth control, including COCs [41]. The study that observed adolescents and women aged 15-34 years suggests that the use of all types of hormonal contraceptives (COCs, progestin-only pills, levonorgestrel vaginal ring, levonorgestrel intrauterine device, and medroxyprogesterone acetate depot) was positively associated with the subsequent use of antidepressants and the clinical diagnosis of depression and that adolescents were more vulnerable to this risk compared to women between 20 and 34 years old [41]. The findings of this study also suggested that using hormonal contraception was subsequently associated with the increased usage of antidepressants. Similarly, research from the same group also identified that the usage of hormonal contraception had a positive association with subsequent suicide attempt and suicide [42]. Another prospective cohort study indicated that teenage patients using oral contraceptives (OC) had a higher rate of exhibiting depressive symptoms and behaviors, including crying, appetite inconsistencies, and fatigue, as compared to those who did not use oral contraceptives [43]. A recent population-based cohort study concluded that oral contraceptive usage, shortly after initiation, is associated with an increased risk of depression in adolescents, as well as in adults, and warrants further research to examine the relationship between COC usage and depression [44].

In a longitudinal study that followed 10 healthy premenopausal women between 16 and 40 years old who started the usage of oral contraceptives from January 2015 to August 2018, the researchers demonstrated no significant change in the gut microbiome diversity or composition but identified marginal changes in the function of the gut microbiome [45]. The participants in the study provided blood and stool samples at three time points: baseline prior to the initiation of OC use and one month and six months after the initiation of OC use. The group, however, did not evaluate the type of COC used in the study. A recent study was carried out to quantify sex differences in GI barrier function at rest and assess whether GI permeability was augmented by COC use [46]. Twenty-seven participants completed the study, which included nine men, nine eumenorrheic women (natural menstrual cycle {MC} group), and nine women taking a monophasic COC (OC group). The authors assessed GI permeability by using a dual sugar absorption technique (the ratio of lactulose-to-rhamnose excretion through urine) and concluded that the ratio was higher in the OC group compared to MC women and men, suggesting that the OC group had the highest intestinal permeability; however, neither permeability nor the release of tumor necrosis factor-alpha (TNF-α) were impacted by the pill cycle [46].

Based on the existing evidence, COC could contribute to changes in bacterial composition and the permeability of the tract, and these changes in the gut have the potential to contribute to mental health complications such as anxiety, depression, and psychiatric disorders. Most recently, a review that explored the same proposition reviewed in this article poses the question of whether the gut-brain axis is one of the components that is being missed when trying to find the relationship between combined oral contraceptives and mental health [3]. While there is evidence, further research is essential to establish the direct role of COCs in the development of mental health complications through their impact on the gut microbiome.

## Conclusions

In this paper, we presented an overview of the literature discussing the effects of COCs on gut microbiome and discussed the effects of dysbiosis on the development of CNS complications and inflammatory bowel diseases. The literature review suggests that rather than the gut-estrogen axis and the gut-brain axis being two separate entities, there may be a combined estrogen-gut-brain axis, which may explain the indirect role of COCs on mood disorders through their influence on the gut microbiome. Research on the role of COCs and mood disorders is still emerging, and based on existing evidence, it is reasonable to say that COCs could play a role in affecting the diversity and richness of microbiome composition and also impact gastrointestinal permeability that could potentially contribute to dysbiosis, inflammation, neurotransmitter imbalance, and the development of mood disorders and other mental health complications. In light of these new developments that show the impact of COCs on women’s health and the vast majority of women who use these contraceptive modalities, it is imperative that further research is warranted to fully understand their health implications.

In conclusion, the introduction of the exogenous sex hormones contained in COCs into the body may have the potential to contribute to the disruption of an individual’s normal endogenous environment and, in turn, the normal bacterial composition within the gut. Alterations to the makeup of the gut microbiome can lead to various health issues, including the development of mood disorders and anxiety. Primary healthcare practitioners must understand the potential role of COCs in precipitating depression and anxiety in women and follow up with their patients in three months to evaluate the need for the substitution or replacement of an existing regimen. Based on the available evidence of COCs with negative mood and depression, caution should be exercised in women with a personal or family history of depression. Further research is warranted to fully understand the association of COCs, estrogen-gut-brain axis, and their impact on the gut microbiome in contributing to the development of depression, anxiety, and other psychiatric conditions.
